# Non-genetic inheritance and the patterns of antagonistic coevolution

**DOI:** 10.1186/1471-2148-12-93

**Published:** 2012-06-21

**Authors:** Rafal Mostowy, Jan Engelstädter, Marcel Salathé

**Affiliations:** 1 Institute for Integrative Biology, ETH Zurich, 8092 Zurich, Switzerland; 2 Department of Infectious Disease Epidemiology, Imperial College, St Mary’s Campus, Norfolk Place, London W2 1PG, UK; 3 Institute for Biogeochemistry and Pollutant Dynamics, ETH Zurich, 8092 Zurich, Switzerland; 4 School of Biological Sciences, The University of Queensland, Brisbane, 4072, QLD, Australia; 5 Department of Biology, Center for Infectious Disease Dynamics, Pennsylvania State University, University Park, 16802, PA, USA

**Keywords:** Host, Parasite, Coevolution, Epigenetic inheritance, Resistance, Red queen dynamics

## Abstract

**Background:**

Antagonistic species interactions can lead to coevolutionary genotype or phenotype frequency oscillations, with important implications for ecological and evolutionary processes. However, direct empirical evidence of such oscillations is rare. The rarity of observations is generally attributed to inherent difficulties of ecological and evolutionary long-term studies, to weak or absent interaction between species, or to the absence of negative frequency-dependence.

**Results:**

Here, we show that another factor – non-genetic inheritance, mediated for example by epigenetic mechanisms – can completely eliminate oscillations in the presence of such negative frequency dependence, even if only a small fraction of offspring are affected. We analytically derive the threshold value of this fraction at which the dynamics change from oscillatory to stable, and investigate how selection, mutation and generation times differences between the two species affect the threshold value. These results strongly suggest that the lack of phenotype frequency oscillations should not be attributed to the lack of strong interactions between antagonistic species.

**Conclusions:**

Given increasing evidence of non-genetic effects on the outcomes of antagonistic species interactions, we suggest that these effects should be incorporated into ecological and evolutionary models of interacting species.

## Background

The phenotypes of species are generally assumed to be adapted to their environment by natural selection. A change in an environment can therefore lead to an evolutionary change in phenotypes as species adapt to new circumstances. Environments comprise both biotic and abiotic elements, and evolutionary change in one species is often driven by evolutionary change in another species. Indeed, ecology is dominated by species interactions such as predation, parasitism, mutualism and competition. If species interactions are antagonistic (i.e., one species benefits at the expense of another), the resulting patterns of adaptation and counter-adaptation can lead to cyclical dynamics typical of predator-prey or host-parasite systems. Understanding the causes and consequences of such fluctuating population dynamics is crucial in a number of biological phenomena, and particularly also in applied fields such as conservation biology and pest management.

The population dynamics of antagonistic species interactions can be captured with well-established models such as the Lotka-Volterra model [1], the Nicholson-Bailey model [2], or the Red Queen model [3]. The Red Queen model stands out as a coevolutionary model because it does not primarily focus on fluctuating population densities, but rather on fluctuating genotype and phenotype frequencies of the interacting species. The causes and consequences of fluctuating genotype and phenotype frequencies in host-parasite, host-parasitoid and predator-prey interactions [4] are increasingly well understood at least in two-species systems, but direct empirical evidence of long-term dynamics is rare [5], not at least because long-term dynamics are inherently difficult to measure [6,7].

Phenotypic adaptations to changing environments need not be driven by natural selection alone. This is because many phenotypes are plastic and can change due to adverse environmental conditions, a property generally referred to as phenotypic plasticity. Interestingly, phenotypic change can be stably transmitted across generations at various levels of specificity. Transgenerational induction of defences has been reported in animals and plants [8-10]. The maternal transfer of antibodies in vertebrates is a well known phenomenon, and in recent years, it has become clear that both vertebrates and invertebrates exhibit transgenerational immunity (see [11,12] and references therein). In the context of host-parasite coevolution, one of the most remarkable demonstrations has been given by [13] who have provided evidence for strain-specific immunity transmitted from mother to offspring in *Daphnia magna* infected with the pathogenic bacteria *Pasteuria ramosa*. Also, transgenerational phenotypic inheritance of virulence traits has been demonstrated in the malaria parasite *Plasmodium falciparum*[14]. In the microbial world, phenotypic switching has been reported both as a direct response to environmental change [15] and as a stochastic event [16] anticipating environmental change, and phenotypic states are often inherited stably across generations [17,18]. For a recent review of non-genetic inheritance and its evolutionary implications see [19].

Our goal here is to understand the effect of non-genetic inheritance on patterns of antagonistic coevolution. We develop a simple model where two species (e.g., host and parasite) are interacting, and each species has two alternative phenotypes. If their phenotypes match, the outcome of the interaction has negative fitness consequences for one species (host) and positive for the other species (parasite). As a result of this, the phenotypes harmed by the interaction may switch to the alternative phenotype in the offspring. We are purposefully ignorant about the nature of the phenotype (e.g., molecular, developmental, behavioral) and about the underlying form of non-genetic inheritance responsible for the phenotype switch in the offspring. In the absence of non-genetic inheritance, this model reduces to the most basic model of antagonistic coevolution exhibiting negative frequency dependence and resulting in the classical Red Queen dynamics (i.e., oscillations of phenotypes). We find that non-genetic inheritance can strongly affect cycling behavior typical of Red Queen dynamics by dampening the phenotype frequency oscillations. To examine this in detail, we derive analytical expressions of the threshold rate at which this elimination occurs.

## Methods

In order to understand how non-genetic inheritance affects the patterns of antagonistic coevolution, we consider a simple, discrete-generation, coevolutionary model of two species *X* and *Y * which interact antagonistically, e.g., hosts and parasites or predators and their preys. Each species is represented as a haploid, single-locus genotype with two possible alleles. The locus can be a genetic factor (gene or genotype) encoding for a given phenotype, or simply a phenotype itself. The two populations, *X* (host or prey) and *Y * (parasite or predator) thus carry two alternative phenotypes, 1 and 2, and the model tracks the frequency of each phenotype in every generation. We assume both populations to be infinitely large and initiate their phenotype frequencies at random. To approach its long-term dynamics, coevolution of *X* and *Y * proceeds for 11000 generations, and only during the last 1000 generations are the measurements taken. At each generation, both species undergo selection and reproduce; the crucial feature of the latter process is the ability to switch phenotypes due to antagonistic interaction.

Antagonistic interactions induce fitness costs on both species: successful interactions come at a cost for species *X* while unsuccessful interactions come at a cost for species *Y *. We assume that successful interaction occurs between the corresponding phenotypes. We denote the frequencies of phenotypes 1 and 2 from species *X* as *x*_1_ and *x*_2_, and phenotype frequencies from species *Y * as *y*_1_and *y*_2_. Only individuals from species *Y * with phenotype 1 successfully attack individuals from species *X* with phenotype 1, and only individuals from species *Y * with phenotype 2 successfully attack individuals from species *X* with phenotype 2. This results in selection against the matching phenotypes in *X* and the non-matching phenotypes in *Y *, such that only a fraction 1−*s*_*X *_and 1−*s*_*Y*_, respectively, survive in the next generation (see Table 1); the frequencies in the two population are changed accordingly. Both species then undergo reproduction, which may involve induced phenotype switching and stochastic phenotype switching. Induced switching is induced by the antagonistic interaction: individuals which are harmed by the interaction transmit the opposite phenotype to the next generation in the following proportions: *α*_*X *_for species *X* and *α*_*Y *_for species *Y *. The phenotype frequencies after selection and induced switching read

(1) $$\begin{array}{lll} x^{\prime } & =\frac{x(1-s_{X}\;y)+\alpha_{X}(1-s_{X})(1-x-y)}{1-s_{X}\left[\text{xy}+(1-x)(1-y)\right]},\; & \;\\ y^{\prime } & =\frac{y[1-s_{Y}\;(1-x)]+\alpha_{Y}(1-s_{Y})(x-y)}{1-s_{Y}\left[x(1-y)+(1-x)y\right]},\; \end{array}$$

where we have assumed that *x*_1_ + *x*_2_ =* y*_1_ + *y*_2_ = 1, *x *=* x*_1_, and *y *=* y*_1_. Equation (1) can be derived in three steps. First, one calculates the proportion of individuals of species *X* which are matched by species *Y *, here equal to *xy*. Second, this proportion of individuals undergoes selection, which means that the frequencies of the matched individuals are multiplied by $(1-s_{X})/\bar{w}$, while the frequencies of the unaffected individuals are multiplied by $1/\bar{w}$, where $\bar{w}$ is the species X mean population fitness. Third, the matched individuals undergo induced switching, meaning that the proportion *α*_*X*_ of them switches to an alternative phenotype. This yields the first equation in (1), and the calculation for species *Y * is analogous. As those individuals which undergo induced switching are also those which have undergone selection, the selection coefficient *s*_*X*_ becomes equivalent to a cost of induced switching, as seen in equation (1) above (see also Discussion).

**Table 1:** **The fitness values resulting from the antagonistic interaction between *X *and *Y ***

**rel. fitness of species*X***	***y***_**1**_	***y***_**2**_	**rel. fitness of species *Y***	***x***_**1**_	***x***_**2**_
*x*_1_	1−*s*_*X*_	1	*y*_1_	1	1−*s*_*Y*_
*x*_2_	1	1−*s*_*X*_	*y*_2_	1−*s*_*Y*_	1

In contrast, stochastic switching occurs independently of antagonistic interactions, and in proportion *μ* in both species *X* and *Y *. Therefore, the frequencies after stochastic switching (and thus after one generation) are given by

(2) $$\begin{array}{ll} x^{\prime \prime }=(1-\mu )x^{\prime }+\mu (1-x^{\prime }) & \;\\ y^{\prime \prime }=(1-\mu )y^{\prime }+\mu (1-y^{\prime }). & \; \end{array}$$

This step can be also interpreted as mutation, and we generally assume that *μ *= 10^−8^ unless mentioned otherwise.

Finally, we allow for asymmetry in the generation time between the two species by defining a parameter *g*, which denotes the number of generations that species *Y * undergoes in a single generation of species *X*. During one generation of *Y *, a fraction 1/*g*of the population *X* is updated according to the equations given above, while the fraction 1−1/*g*remains unchanged. This process is then repeated *g* times, and the resulting frequencies *x*^*′′*^ and *y*^*′′ *^yield the phenotype frequencies after an entire generation of species *X*[20]. By default, we assume *g *= 1 unless mentioned otherwise.

## Results

**Figure 1:**
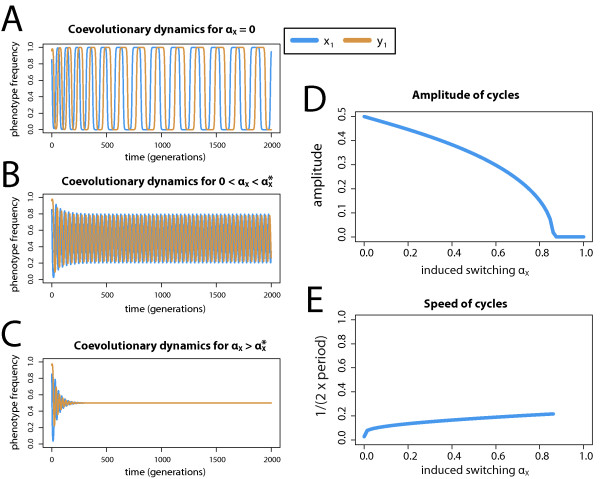
**Impact of induced phenotypic switching on the cyclic phenotype frequency dynamics.** (**A**): Coevolutionary dynamics between antagonistic phenotypes are predicted to continue indefinitely when no induced switching occurs due to time-lagged, negative frequency dependent selection (*α*_*X *_= 0). (**B**): When induced switching occurs (here in only in species *X*, thus *α*_*Y *_= 0) at a low rate ( $0<\alpha_{X}<\alpha_{X}^{*}$), allele frequency cycles persist in time but at an altered amplitude and speed. (**C**): When the switching rate exceeds a threshold value $\alpha_{X}^{*}$, cycles begin to dampen and reach a stable equilibrium. (**D-E**): Increased levels of induced switching also decrease the amplitude and increase the speed of the cycles. Note that $\alpha_{X}^{*}$ in panels A-C and the one in panels D-F is different because of different selection coefficients used; the relation between the strength of selection and the persistence of cycles is examined in detail in the subsequent figure. The following parameter values were used: (A-C) *s*_*X *_=* s*_*Y *_= 0.3, (D-E) *s*_*X *_=* s*_*Y *_= 0.65; (A) *α*_*X *_= 0, (B) *α*_*X *_= 0.03, (C) *α*_*X *_= 0.1; In all panels we used *α*_*Y *_= 0. Period is defined as a number of generations during which the phenotype frequency cycles around to its original value.

It is generally expected that antagonistic interactions can result in cyclic allele frequency dynamics, reflecting a continuing arms race between the two species [21]. In the absence of induced phenotypic switching (*α*_*X *_=* α*_*Y *_= 0) our model reveals such a pattern (see Figure 1A). In this situation, if the common phenotype of species *X* (say phenotype 1) is more likely to interact antagonistically with the corresponding matching phenotype of species *Y * (phenotype 1), then another phenotype 2 of species *X* has a selective advantage causing a gradual increase of the frequency *x*_2_ and a decrease of the frequency *x*_1_. Such change will in turn drive the frequency change in species *Y * by selecting for phenotype 2, causing *y*_2_ to increase, and so on. These oscillations are, in the absence of random genetic drift and mutation, expected to continue indefinitely, otherwise fixation or extinction of one of the two phenotypes occurs [22].

Consider now a situation where induced phenotypic switching is possible in a single species. Figure 1B-C shows the impact of such a process on the frequency dynamics between species *X* and *Y *. We see that as the switching rate increases in species *X* (*α*_*X *_> 0, *α*_*Y *_= 0), the cycles become faster and of lower amplitude, eventually leading to a stable state (*x*^∗^,*y*^∗^) = (1/2,1/2); (Figure 1C). This happens when the switching rate *α*_*X*_ exceeds a certain threshold value, $\alpha_{X}^{*}$, such that when $\alpha_{X}<\alpha_{X}^{*}$ the cycles are maintained (even though with altered amplitude and frequency), and when $\alpha_{X}>\alpha_{X}^{*}$, cycles dampen and reach a stable equilibrium. The changes in speed and amplitude of cycles are directly measured in Figure 1D-E, and show that as *α*_*X *_increases the amplitude gradually decreases to zero and the speed increases until the cycles disappear. This already illustrates that induced switching can fundamentally affect the oscillatory dynamics in the system.

In order to examine the persistence of cyclic dynamics in more detail, we derive an analytical expression for the stability of the cyclic behaviour as a function of *α*_*X*_, *α*_*Y*_, *s*_*X*_, and *s*_*Y*_.

In the case of the model considered here, the stability requires that *x*^*′′ *^=* x* and *y*^*′′ *^=* y*, where *x*^*′′ *^≡* x*(*t* + 1), *x *≡* x*(*t*), *y*^*′′ *^≡* y*(*t* + 1), and *y *≡* y*(*t*). It can be shown that the four trivial equilibria of this system are (*x*^∗^,*y*^∗^) = (0,0), (*x*^∗^,*y*^∗^) = (0,1), (*x*^∗^,*y*^∗^) = (1,0), (*x*^∗^,*y*^∗^) = (1,1), and that (*x*^∗^,*y*^∗^) = (1/2,1/2) is a non-trivial equilibrium with the Jacobian of the form

(3) $$\;\;\;J{|}_{\left(1/2,1/2\right)}=(1-2\mu )\left(\begin{array}{c} 1-\frac{2(1-s_{X})\alpha_{X}}{2-s_{X}}\;-\frac{s_{X}+2\alpha_{X}-2\alpha_{X}s_{X}}{2-s_{X}}\\ \frac{s_{Y}+2\alpha_{Y}-2\alpha_{Y}s_{Y}}{2-s_{Y}}\;\;1-\frac{2(1-s_{Y})\alpha_{Y}}{2-s_{Y}} \end{array}\right)\;.$$

The corresponding eigenvalues are

(4) $$\lambda_{\pm }=(1-2\mu )\left(a\pm \frac{\sqrt{b-2c}}{\left(2-s_{X})(2-s_{Y}\right)}\right),$$

where

(5) $$\begin{array}{lll} a & =1-\frac{\alpha_{X}(1-s_{X})}{2-s_{X}}-\frac{\alpha_{Y}(1-s_{Y})}{2-s_{Y}},\; & \;\\ b & ={\left(2-s_{Y}\right)}^{2}{\left(1-s_{X}\right)}^{2}\alpha_{X}^{2}+{\left(2-s_{X}\right)}^{2}{\left(1-s_{Y}\right)}^{2}\alpha_{Y}^{2},\; & \;\\ c & =(2-s_{X})(2-s_{Y})\left[s_{X}s_{Y}/2+s_{X}(1-s_{Y})\alpha_{Y}\right)\; & \;\\ & \left(\;+\;s_{Y}(1-s_{X})\alpha_{X}+3(1-s_{X})(1-s_{Y})\alpha_{X}\alpha_{Y}\right].\; & \; \end{array}$$

This has been derived under the assumptions of 0 ≤* s*_*X*_,*s*_*Y *_≤ 1, 0 ≤* α*_*X*_,*α*_*Y *_≤ 1, and 0 ≤* μ *< 0.5. The condition for the stability at (*x*^∗^,*y*^∗^) = (1/2,1/2) requires that the absolute value of both eigenvalues be smaller than one, or

(6) $$|\lambda_{+}|<1\;\mathrm{and}\;|\lambda_{-}|<1.$$

The inequality (5) yields constraints on the values of *α*_*X*_ and *α*_*Y*_ for which, given *s*_*X *_and *s*_*Y*_, the equilibrium (*x*^∗^,*y*^∗^) = (1/2,1/2) is unstable, resulting in persisting phenotype frequency oscillations, or for which the equilibrium is stable, resulting in the cessation of the cycles.

The induced switching values for which the stability of the system is lost or regained can be calculated analytically for special cases of the stability condition (5), and otherwise either numerically or estimated from the simulation results. For example, when *α*_*X *_=* α*_*Y *_=* α*, *s*_*X *_=* s*_*Y *_=* s*, and *μ *= 0, the condition (5) is equivalent to

(7) $$\;\;\;\;\;\frac{2}{{\left(2-s\right)}^{2}}\left[2-4\alpha {\left(1-s\right)}^{2}+4\alpha^{2}{\left(1-s\right)}^{2}-(2-s)s\right]<1.$$

It can be shown that the left-hand side of the inequality (6) is an increasing function of *s* in the range of *α* for 0 ≤* s*≤ 1, and 0 ≤* α *≤ 1. Therefore, an increasing selection strength will tend to induce cycles rather than destroy them. It can be also shown that the left-hand side of inequality (6) is an decreasing function of *α* for 0 ≤* α *< 0.5, and an increasing function of *α* for 0.5 <* α *≤ 1. Therefore, as induced switching *α* increases, cycles can be lost at low values of *α*and regained at high values of *α*. The inequality (6) can also be expressed as

(8) $$\;\;\;\;\;\alpha >\frac{1}{2}-\sqrt{\frac{2-(4-s)s}{8{\left(1-s\right)}^{2}}}\;\text{and}\;\alpha <\frac{1}{2}+\sqrt{\frac{2-(4-s)s}{8{\left(1-s\right)}^{2}}},$$

which allows a precise calculation of the threshold levels of induced switching at which cycles disappear and reappear in this particular example.

**Figure 2:**
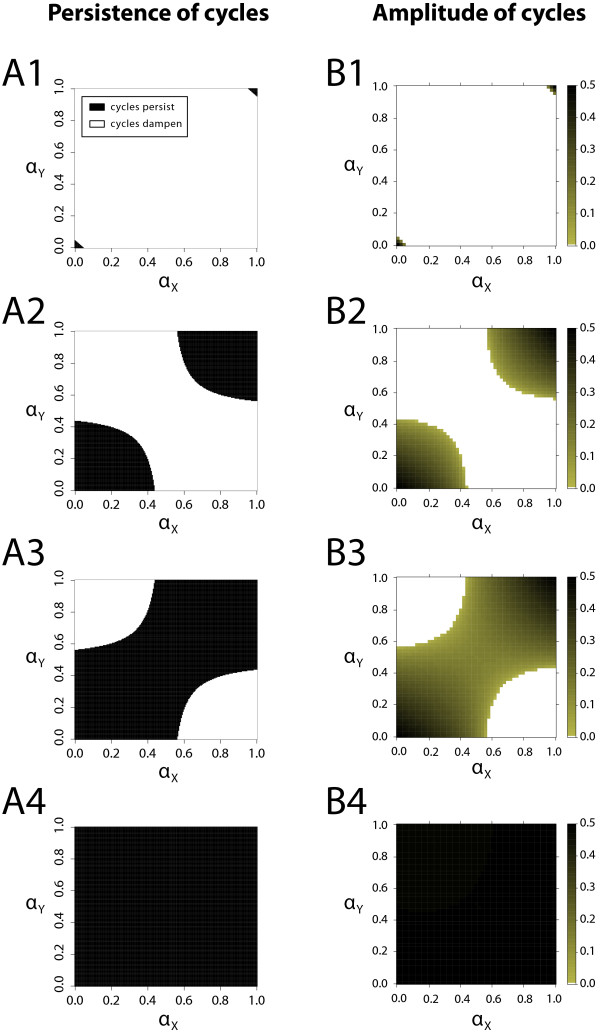
**Impact of the strength of selection on the persistence of cyclic phenotype frequency dynamics (A1–A4): Persistence of phenotype oscillations (black regions) for increased selection coefficients based on analytical predictions.** When selection is weak (A1) cycles will continue indefinitely only for minimal or maximal levels of induced switching in both species. As selection becomes stronger (A2–A3), cyclic dynamics become more difficult to destroy and persist at higher levels of induced switching. For exceptionally high values of selection coefficients (A4), allele frequency oscillations will become unaffected by induced switching, including their speed and amplitude. As explained in the main text, the nature of cycles for minimal and maximal values of induced switching is different because of being driven by different evolutionary forces. (**B1–B4**): Numerical calculation of the amplitude of cycles from computer simulations confirms the analytical calculations in panels A1-A4. It also reveals that the amplitude of cycles decreases as the cycles reemerge at high values of induced switching in both species *X* and *Y *. The following parameter values were used: (A1,B1) *s*_*X *_=* s*_*Y *_= 0.30, (A2,B2) *s*_*X *_=* s*_*Y *_= 0.57, (A3,B3) *s*_*X *_=* s*_*Y *_= 0.60, (A4,B4) *s*_*X *_=* s*_*Y *_= 0.90; panels A1-A4 were made in resolution 101×101; in panels B1-B4 were made in resolution 51×51, and cycles of amplitude 0.5×10^−2^ where considered non-existent.

To examine the persistence of cycles for a general case of *α*_*X *_≠* α*_*Y*_, we solve the relation (5) numerically and compare it with the simulation results. Figure 2A shows the combinations of induced switching values *α*_*X *_and *α*_*Y *_for which oscillations dampen, with different combinations of selection coefficients, based on the stability condition (5). Figure 2B shows the analogous results which are extracted from the simulations. The presence or absence of allele frequency cycles in simulations is measured by the amplitude of the cycles after 11000. We define the presence of cycles if such amplitude exceeds the threshold of 5×10^−2^, and otherwise we consider the cycles to be absent. A comparison between Figure 2A and 2B shows that simulation results and analytical predictions are in good accordance, and we see this in all examined regions of the parameter space. We also see that the results are largely independent of the value of the threshold used to measure the presence of allele cycles, provided the simulations run long enough.

The results in Figure 2 illustrate a few important points. First, when both species undergo induced switching, lower rates of switching are needed to destroy the cyclic behaviour (cf. Figure 2A2). Second, as shown above, when the two species switch phenotypes at the same rate *α *=* α*_*X *_=* α*_*Y*_, the cycles can reemerge as *α *→ 1. Finally, as also shown above, an increased selection pressure makes the cyclic dynamics more robust to higher levels of induced switching. In fact, as our calculations reveal, this dependence is so strong that for selection coefficient of 0.1 in both species as little as 0.5% of induced switching in species *X* is enough to eradicate the cyclic dynamics, while for selection coefficient of 0.9 induced switching will never dampen the cycles. The results for asymmetric selection coefficients are qualitatively identical.

Interestingly, the nature of cycles for low and high levels of induced switching is very different. In the case of *α*_*X *_=* α*_*Y *_= 0, the oscillatory behaviour will persist due to time-delayed negative frequency-dependent selection (being rare is advantageous, being common is disadvantageous), whereas for *α*_*X *_=* α*_*Y *_= 1 oscillations will occur even in the absence of a selective force. The reason for this is that the latter situation represents the case where the phenotype frequency of one species in the next generation will be fully determined by the frequency of the phenotype of the other species. This will result in one species being constantly adapted to the other species population in the previous generation. However, since the other species does exactly the same, the two species will constantly cross-react even in the absence of any evolutionary force, leading to oscillatory “mirror dynamics” (see Discussion). Interestingly, in the parameter regime where these dynamics are dominant, increasing induced switching increases the amplitude of the allele cycles, while in the parameter regime where selection-induced cycles are dominant, increasing induced switching decreases the amplitude of the allele cycle, as seen in Figure 2B1-B4.

**Figure 3:**
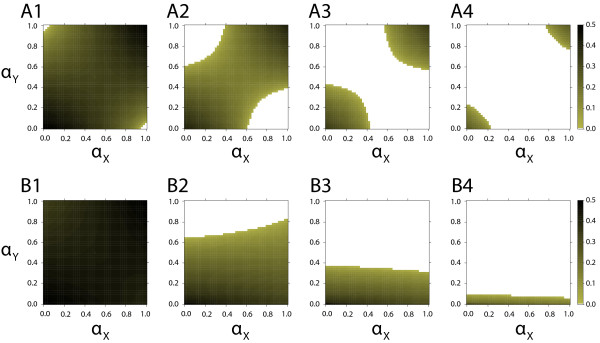
**Impact of stochastic phenotypic switching and asymmetric generation times on the persistence of phenotype oscillations.** (**A1–A4**): Persistence and amplitude of cycles for different stochastic switching values. Subsequent panels show the results for increased values of *μ*, illustrating that high levels of stochastic switching can also destroy the persistence of the cyclic phenotype frequency dynamics. (**B1-B4**): Persistence and amplitude of cycles for different asymmetric generation times between species *X* and *Y *. Subsequent panels show the results for increased values of *g* (number of *Y * generations per *X* generation). Rapid adaptation of one species also destroys cyclic dynamics, although the impact on cycles at high values of induces switching (especially *α*_*X*_) is much less prominent. The following parameter values were used: (A1-A4) *s*_*X *_=* s*_*Y *_= 0.66, (B1-B4) *s*_*X *_=* s*_*Y *_= 0.75; (A1) *μ *= 10^−8^, (A2) *μ *= 2×10^−2^, (A3) *μ *= 3×10^−2^, (A4) *μ *= 4×10^−2^; (B1) *g *= 1, (B2) *g *= 2, (B3) *g *= 3, (B4) *g *= 10; all panels were produced in resolution 51×51, and cycles of amplitude 0.5×10^−2^where considered non-existent.

Stochastic switching can also destroy cyclic frequency dynamics. This is illustrated in Figure 3A1-A4. As the stochastic switching rate *μ*increases, the parameter space with persistent phenotypic cycles shrinks down. This result is not surprising. Mathematically speaking, the absolute value of eigenvalues (4) decreases as *μ*increases, and therefore the stable state can be sometimes reached for high values of *μ*. This argument has been formulated some time ago in earlier theoretical host-parasite studies when discussing the potential impact of genetic mutation on antagonistic coevolution (see e.g., [23] and references therein). However, as genetic mutation rates are usually thought to be small (with some notable exceptions), they are typically not expected to cause any dampening effects in natural populations. Stochastic switching rates, however, can be orders of magnitude higher [16], and therefore the disappearance of cycles due to stochastic switching is likely to be more pervasive in nature than the disappearance of cycles due to genetic mutation.

Finally, we examined the effect of asymmetric generation times between species *X* and *Y *, a situation that is certainly to be expected in host-parasite systems, and not uncommon in predator-prey systems. The results, shown in Figure 3B1-B4, illustrate that an increased speed of evolution of species *Y * again makes the cyclic dynamics more sensitive to increased levels of induced switching. Interestingly, in this case the oscillatory “mirror dynamics” described above do not emerge for very high values of *α*_*X*_ and *α*_*Y*_. This is because when one of the species evolves faster (here *Y *), the symmetry of these dynamics is violated: species *Y * will always react more quickly, thereby immediately adapting to the other species. Furthermore, induced switching in the species which adapts more slowly (here *X*) has now a minor impact on the phenotype frequency dynamics observed in the model.

## Discussion

Antagonistic coevolution is pervasive in nature, and oscillatory dynamics are generally thought to be one of its key signatures. The stability of this pattern is of fundamental importance in biology because the dynamics of phenotypes and genotypes are central to evolutionary and ecological processes. Furthermore, the absence of oscillations could be interpreted as the absence of an antagonistic interaction. We have shown here that in a simple model of antagonistic coevolution between two species, phenotypic switching – transmitted to the next generation through non-genetic inheritance – can have a dramatic effect on the patterns of antagonistic coevolution. Minimal levels of induced phenotypic switching can completely eliminate oscillatory dynamics and result in stable frequencies. This therefore suggests that even in the presence of strong links between the two species (i.e., strong selection, high specificity, etc.), antagonistic coevolution need not result in fluctuations of genotypes and phenotypes.

We have identified three parameters that affect the threshold level of induced switching at which cycles disappear. The first is the strength of selection in an antagonistic species interaction. For the threshold level to be high, both species need to suffer large fitness costs, to the extent that when selection is strong enough cycles will never be affected. Parasites may indeed pay such costs because their reproduction often depends on a successful antagonistic interaction with a host (see e.g., [24]). On the other hand, while both hosts and predators suffer fitness costs from being infected, or not being able to predate, their costs are arguably much lower. Second, an increase in *g*, the number of generations of the faster evolving species (e.g., the parasite) per generation of the other species (e.g., the host), typically reduces that threshold value. This is particularly relevant in the case of microparasites whose generation times can be many orders of magnitude shorter than that of their hosts. Finally, stochastic events affecting phenotypic switching can also reduce the threshold value. As stochastic switching events are increasingly being discovered in the microbial world, this effect might again be most relevant in the case of host-parasite interactions.

What makes the cycles disappear? Fundamentally, cycles depend on time-lagged, negative frequency-dependent selection (see e.g., [25]). Any factor that acts to reduce the time-lag will act to reduce the amplitudes of cycles. In the absence of induced phenotypic switching, the speed at which the rare phenotype with a fitness advantage will increase in frequency depends on the strength of the antagonistic interaction. Lower fitness costs, higher discrepancy in generation times (i.e., higher *g*) and higher mutation rates all act to reduce the realised strength of interaction. For example, fast evolution in one species can lead to dampened cycles, masking interactions such that even though two species might be tightly linked (i.e., under strong selective pressure), the realized strength of interaction is low [26]. In the presence of induced phenotypic switching, there is limited scope for selection to reduce the frequency of the disadvantaged (common) phenotype; for example when induced switching occurs, counter-adaptation occurs instantly at rate *α*, without the action of natural selection.

**Figure 4:**
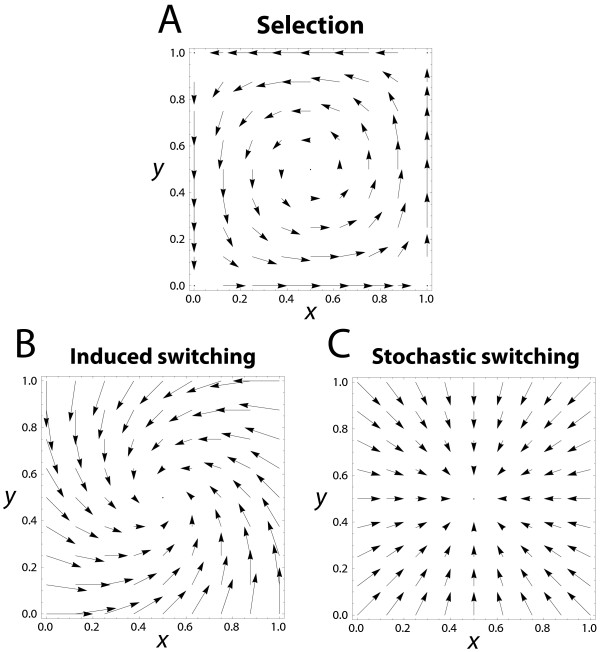
**Impact of the three evolutionary forces of our model on the change in phenotype frequency *x *and *y *during a single time step (one generation), as given by equations (**1**), and (**2**).** The beginning and end of each arrow marks the phenotype frequencies before and after the respective step. As can be seen, selection tends to produce oscillations, whereas both induced and stochastic phenotypic switching tend to dampen these oscillation. Parameters used are (**A**) *s*_*X *_=* s*_*Y *_= 0.5, (**B**) *α*_*X *_=* α*_*Y *_= 0.2, and (**C**) *μ *= 0.1.

A more formal way to describe this phenomenon is to realize that under selection, the change of phenotype frequency in a single generation in one species, say *X*, depends on the variance of phenotype frequencies, *x*(1−*x*). This leads to a certain inertia characteristic of natural selection that produces the time-lags in counter-adaptation and thus the cyclical dynamics. Imagine a very common phenotype of *X* that is confronted with its equally common matching phenotype of *Y *, reducing its fitness. While this matching is obviously detrimental to this phenotype, the change in *x* in the next generation will be relatively small because individuals with this phenotype are mostly competing against individuals of the same phenotype (see upper right and lower left corners of Figure 4A). Under induced switching, however, this is not the case: under the same scenario, the change in *x* does not depend on the variance in phenotypes frequencies: a proportion *α* of the common *X* phenotype that is matched by *Y * will immediately switch to the other phenotype in the next generation (which is why the arrows are so much longer in the corners of Figure 4B). As a consequence, the time-lag will be reduced and oscillations of phenotype frequencies *x* will gradually vanish. Similarly, high levels of stochastic switching will destroy oscillations in phenotype frequencies (Figure 4C).

Overall, one of the most striking findings of this study is just how little phenotypic switching, especially interaction-induced, is necessary to completely eliminate cycles. One is tempted to speculate that such a process could be one of the reasons why evidence of dynamic polymorphisms is so rare, apart from the fact that long-term observations are difficult [7]. However, there are a number of caveats to consider. First, some antagonistic systems are characterised by strong selection [24], in which case we would expect that cycles would be maintained even in the presence of induced phenotypic switching. Second, evidence for induced phenotype switching as envisioned in this model is still rare, despite the fact that the number of demonstrations of strain- or pathogen-specific immunity has been steadily increasing. Third, not every type of induced switching fits the implementation in our model. For example, maternal transfer of antibodies can make the offspring resistant to a pathogen strain encountered by the mother, but it does not come at the cost of becoming susceptible to another strain. However, such a tradeoff assumption is necessary for oscillations to appear in the first place – the model simply argues that if these tradeoffs do exist such that oscillations could be expected all else being equal, then phenotypic switching can dampen the oscillations altogether. Fourth, to what extent phenotype switching is stable across generations is currently largely unknown, and its adaptive value is an open question as well. Fifth, antagonistic fitness interactions are often resulting in fluctuating population densities, which may in turn affect themselves evolutionary dynamics [26-30]. In order to understand the nature of the dynamics of phenotype frequency oscillations, we have purposefully ignored such population dynamics. Furthermore, how these results extend to complex communities of multiple species currently remains unknown. Finally, costs of induced switching may further reduce its dampening effects, provided that these costs are paid only by those individuals who are actually switching. Since we assume that only individuals affected by the interaction transmit the opposite phenotype, the impact of such costs can by easily calculated by multiplying relative fitness coefficient 1−*s* by 1−*c*, where *c* is the cost of switching. In the case of species *X*, the selection coefficient in eq. (1), *s*_*X*_, would by substituted by *s*_*X*_ + *c*−*s*_*X*_*c*. In contrast, in the presence of a general cost of maintaining a sensory mechanism for an antagonistic interaction, every individual would pay the same cost, and relative fitness would not be affected.

One of the important assumptions of this study is that the model underlying the antagonistic interaction is of a ‘matching-alleles’ type. Such a model is mostly applicable in the case of hosts with a specific immune system, and antigenic parasites, which have to specifically match the host in order to infect it. By contrast, interactions in many plant-pathogen systems are usually thought to be of a ‘gene-for-gene’ type, where a host needs to recognise specific ‘effectors’ of the parasite in order to launch its defence [31]. In spite of this difference, the implications of this study bear similarity to the studies of plant-pathogen models, where the conditions for the persistence of oscillatory dynamics and polymorphisms were thoroughly investigated. In particular, it has been previously noted that uncoupling of host and parasite life cycles in time or space can lead to a stabilization of allele cycles [32]. One good example is a high level of polycyclicity in a parasite life cycle, which was shown to induce stable polymorphism over time [33], in analogy to the results of our study (cf., Figure 3B). Analogously, high mutation rates can lead to stable equilibria of allele frequencies in plant-pathogen systems [34,35]. Altogether, the analogies between the ‘matching allele’-based systems and the ‘gene-for-gene’-based systems point to the importance of empirical studies of non-genetic inheritance in both plant-pathogen as well as animal-parasite systems.

## Conclusion

Environmentally induced phenotypic change that is stable across generations has recently been demonstrated in a number of cases, many of them involving stable epigenetic modifications [36,37]. Given the recent advances in this field, we expect many more demonstrations of these phenomena, and we see no obvious reason why they should not be observed in the realm of antagonistic interactions, especially since all species are likely to suffer severe fitness consequences if they are at the losing end of these interactions.

## Competing interests

The authors declare no competing interests.

## Authors’ contributions

RM conceived and designed the study, carried out the simulations, analysed the model and the results, and drafted the manuscript. JE analysed the results, and helped to draft the manuscript. MS conceived and designed the study, analysed the model and the results, and drafted the manuscript. All authors read and approved the final manuscript.

## References

[B1] May RM Stability and Complexity in Model Ecosystems 2001 Princeton, NJ: Princeton University Press

[B2] Hassell MP Comins HN May RM Spatial structure and chaos in insect population dynamics Nature 1991 353 6341 255 258

[B3] Hamilton WD Sex versus non-sex versus parasite Oikos 1980 35 2 282 290

[B4] Nuismer SL Thompson JN Coevolutionary alternation in antagonistic interactions Evolution 2006 60 11 2207 2217 17236414

[B5] Schmid-Hempel P Evolutionary Parasitology: The Integrated Study of Infections, Immunology, Ecology, and Genetics 2011 New York: Oxford University Press

[B6] Abrams PA The evolution of predator-prey interactions: theory and evidence Annu Rev Ecol Syst 2000 31 79 105

[B7] Little TJ The evolutionary significance of parasitism: do parasite-driven genetic dynamics occur ex silico? J Evol Biol 2002 15 1 9

[B8] Agrawal AA Laforsch C Tollrian R Transgenerational induction of defences in animals and plants Nature 1999 401 6748 60 63

[B9] Agrawal AA Phenotypic plasticity in the interactions and evolution of species Science 2001 294 5541 321 326 11598291 10.1126/science.1060701

[B10] Poulin R Thomas F Epigenetic effects of infection on the phenotype of host offspring: parasites reaching across host generations Oikos 2008 117 3 331 335

[B11] Hasselquist D Nilsson JÅ Maternal transfer of antibodies in vertebrates: trans-generational effects on offspring immunity Phil Trans R Soc B 2009 364 1513 51 60 18926976 10.1098/rstb.2008.0137 PMC2666691

[B12] Schulenburg H Kurtz J Moret Y Siva-Jothy MT Introduction. ecological immunology Phil Trans R Soc B 2009 364 1513 3 14 18926970 10.1098/rstb.2008.0249 PMC2666701

[B13] Little TJ O’Connor B Colegrave N Watt K Read AF Maternal transfer of strain-specific immunity in an invertebrate Curr Biol 2003 13 6 489 492 12646131 10.1016/s0960-9822(03)00163-5

[B14] Lopez-Rubio JJ Gontijo AM Nunes MC Issar N Hernandez Rivas R Scherf A 5? flanking region of var genes nucleate histone modification patterns linked to phenotypic inheritance of virulence traits in malaria parasites Mol Microbiol 2007 66 6 1296 1305 18028313 10.1111/j.1365-2958.2007.06009.x PMC2228885

[B15] Miller C Thomsen LE Gaggero C Mosseri R Ingmer H Cohen SN SOS response induction by beta-lactams and bacterial defense against antibiotic lethality Science 2004 305 5690 1629 1631 15308764 10.1126/science.1101630

[B16] Acar M Mettetal JT van Oudenaarden A Stochastic switching as a survival strategy in fluctuating environments Nat Genet 2008 40 4 471 475 18362885 10.1038/ng.110

[B17] Lim HN van Oudenaarden A A multistep epigenetic switch enables the stable inheritance of DNA methylation states Nat Genet 2007 39 2 269 275 17220888 10.1038/ng1956

[B18] Rando OJ Verstrepen KJ Timescales of genetic and epigenetic inheritance Cell 2007 128 4 655 668 17320504 10.1016/j.cell.2007.01.023

[B19] Bonduriansky R Day T Nongenetic inheritance and its evolutionary implications Annu Rev Ecol Evol Syst 2009 40 103 125

[B20] Kouyos RD Salathé M Bonhoeffer S The Red Queen and the persistence of linkage-disequilibrium oscillations in finite and infinite populations BMC Evol Biol 2007 7 211 17986336 10.1186/1471-2148-7-211 PMC2198919

[B21] Woolhouse MEJ Webster JP Domingo E Charlesworth B Levin BR Biological and biomedical implications of the co-evolution of pathogens and their hosts Nat Genet 2002 32 4 569 577 12457190 10.1038/ng1202-569

[B22] Parker MA Pathogens and sex in plants Evol Ecol 1994 8 5 560 584

[B23] Nee S Antagonistic co-evolution and the evolution of genotypic randomization J Theor Biol 1989 140 4 499 518 2615403 10.1016/s0022-5193(89)80111-0

[B24] King KC Jokela J Lively CM Trematode parasites infect or die in snail hosts Biol Lett 2011 7 2 265 268 20961880 10.1098/rsbl.2010.0857 PMC3061183

[B25] Bell G The Masterpiece of Nature: The Evolution and Genetics of Sexuality 1982 Berkeley: Croom Helm and University of California Press

[B26] Yoshida T Ellner SP Jones LE Bohannan BJM Lenski RE Hairston NG Cryptic population dynamics: rapid evolution masks trophic interactions PLoS Biol 2007 5 9 e235 17803356 10.1371/journal.pbio.0050235 PMC1964773

[B27] Abrams PA The effects of switching behavior on the evolutionary diversification of generalist consumers Am Nat 2006 168 5 645 659 17080363 10.1086/507878

[B28] Mougi A Kishida O Reciprocal phenotypic plasticity can lead to stable predator-prey interaction J Anim Ecol 2009 78 6 1172 1181 19622080 10.1111/j.1365-2656.2009.01600.x

[B29] Mougi A Iwasa Y Evolution towards oscillation or stability in a predator-prey system Proc Biol Sci 2010 277 1697 3163 3171 20504808 10.1098/rspb.2010.0691 PMC2982064

[B30] Mougi A Kishida O Iwasa Y Coevolution of phenotypic plasticity in predator and prey: why are inducible offenses rarer than inducible defenses? Evolution 2011 65 4 1079 1087 21062279 10.1111/j.1558-5646.2010.01187.x

[B31] Thompson JN Burdon JJ Gene-for-gene coevolution between plants and parasites Nature 1992 360 6400 121 125

[B32] Brown JK Tellier A Plant-parasite coevolution: bridging the gap between genetics and ecology Annu Rev Phytopathol 2011 49 345 367 21513455 10.1146/annurev-phyto-072910-095301

[B33] Tellier A Brown JK Stability of genetic polymorphism in host-parasite interactions Proc Biol Sci 2007 274 1611 809 817 17251091 10.1098/rspb.2006.0281 PMC2093977

[B34] Kirby GC Burdon JJ Effects of mutation and random drift on leonard’s gene-for-gene coevolution model Phytopathology 1997 87 5 488 493 18945102 10.1094/PHYTO.1997.87.5.488

[B35] Segarra J Stable polymorphisms in a two-locus gene-for-gene system Phytopathology 2005 95 7 728 736 18943003 10.1094/PHYTO-95-0728

[B36] Kaufmann BB Yang Q Mettetal JT Van Oudenaarden A Heritable stochastic switching revealed by single-cell genealogy PLoS Biol 2007 5 9 e239 17803359 10.1371/journal.pbio.0050239 PMC1964776

[B37] Jablonka E Raz G Transgenerational epigenetic inheritance: prevalence, mechanisms, and implications for the study of heredity and evolution Q Rev Biol 2009 84 2 131 176 19606595 10.1086/598822

